# Compliance With the Cigarettes and Other Tobacco Products Act (COTPA) 2003 in Deoghar, Jharkhand: A Cross-Sectional Study in a Pilgrimage Town of Eastern India

**DOI:** 10.7759/cureus.95854

**Published:** 2025-10-31

**Authors:** Vinayagamoorthy Venugopal, Ujjwal Kumar, G Jahnavi, Santanu Nath, Arshad Ayub, Bijit Biswas, Venkata Lakshmi Narasimha, Benazir Alam, Niwedita Jha, Saurabh Varshney

**Affiliations:** 1 Community and Family Medicine, All India Institute of Medical Sciences, Deoghar, IND; 2 Vital Strategies Tobacco Control Project, All India Institute of Medical Sciences, Deoghar, IND; 3 Psychiatry, All India Institute of Medical Sciences, Deoghar, IND; 4 Otolaryngology, All India Institute of Medical Sciences, Deoghar, IND

**Keywords:** compliance, india, legislation & jurisprudence, public health, smoking, tobacco control

## Abstract

Background: India faces a mounting public health burden from tobacco use, with over a million annual deaths. The Cigarettes and Other Tobacco Products Act (COTPA) 2003 regulates tobacco control, but compliance in pilgrimage-based semi-urban towns remains under-researched. This study assessed COTPA adherence in public settings of Deoghar, Jharkhand, India.

Methods: A cross-sectional observational survey was conducted at 50 purposively selected public sites, educational institutions, health facilities, religious places, public transport hubs, and libraries using a structured checklist. Compliance with COTPA sections 4 (smoke-free public places), 5 (advertising ban), 6a (sale to/by minors), 6b (sale near educational institutions), and 7 to 9 (packaging and labeling) was evaluated. Data were summarized as frequencies and percentages; 95% confidence intervals were estimated for non-compliance.

Results: Overall non-compliance was highest for section 4 (26/50, 52.0%) and section 6b (10/24, 41.7%). Smoking occurred frequently in transport hubs, libraries, and religious places. Violations regarding sales to minors were present in 8/50 (16.0%) of sites, and tobacco sales within 100 yards of schools in 10/24 (41.7%) of educational institutions. Section 5 violations (advertisement) were seen in 3/50 (6.0%) of sites. Of the 28 sites that sold smokeless tobacco products, 26/28 (92.8%) had no warning pictures printed on the package, in violation of section 7(1). Sections 7.5 (1/28, 3.6%), 8.1 (0/28, 0.0%), and 9.2 (0/28, 0.0%) showed low or no violations.

Conclusion: The enforcement of the COTPA in Deoghar is suboptimal, especially in high-footfall and community settings. Enhanced monitoring, awareness drives, and integration of tobacco control into existing health programs are needed to promote sustained compliance.

## Introduction

Tobacco use remains a leading global public health challenge, responsible for over 8 million deaths annually, including more than 1.2 million from secondhand smoke [1]. In India, tobacco contributes to over one million preventable deaths each year, with 28.6% of adults using tobacco in some form [2,3]. The economic burden of tobacco far exceeds the revenue generated from its taxation, highlighting the critical need for effective regulation and enforcement [4].

In response, India enacted the Cigarettes and Other Tobacco Products Act (COTPA) in 2003, in line with its ratification of the WHO Framework Convention on Tobacco Control (FCTC) in 2004 [5]. The Act regulates smoking in public places, bans tobacco advertising, mandates appropriate packaging and labeling, restricts sales to minors, and prohibits sales near educational institutions. Despite these provisions, adherence remains inconsistent across regions. Studies in urban centers such as Punjab, Bengaluru, and Mumbai report variable compliance, with violations often observed near schools and inadequate display of statutory warnings [6-10].

Tier-II towns and religious destinations, which experience high population density and seasonal influxes of visitors, have received limited attention. Deoghar, a prominent pilgrimage town in Jharkhand, exemplifies such a setting, especially during the Shravan Mela, when large crowds converge around commercial establishments and educational institutions. Field observations suggest widespread open tobacco sales, passive advertising, and inadequate warning displays.

The present study aims to systematically assess compliance with the COTPA in public places across Deoghar town, Jharkhand, India, and to identify areas requiring enforcement and awareness. The specific objectives were to determine adherence to Section 4, evaluate compliance with Section 5, investigate violations of Section 6, verify compliance with Sections 7-9, and identify patterns of non-compliance across different categories of locations. This study was undertaken as part of a tobacco control project funded by Vital Strategies, which sought to strengthen commitment to tobacco control by fostering greater participation of medical institutions in countering tobacco industry interference. Vital Strategies is a global health organization that works to reduce tobacco use and interference through policy reform, public awareness, and partnerships, supported by the Bloomberg Initiative.

## Materials and methods

Study setting and design

This cross-sectional observational study was conducted to assess compliance with key provisions of the COTPA in Deoghar town, Jharkhand, India. Deoghar is a prominent religious and cultural center that attracts large crowds during festivals and is home to multiple educational institutions, government offices, markets, healthcare facilities, and public transport hubs. To capture a representative picture of compliance in this semi-urban, pilgrimage-based setting, surveys were conducted across diverse public places.

This study was approved by the Institutional Ethics Committee of All India Institute of Medical Sciences (AIIMS), Deoghar (approval no. 2022-74-EMP-02). The present study was part of a broader tobacco control project. Participation was voluntary, and informed consent was obtained online from all individuals before data collection. Confidentiality and anonymity of participants were safeguarded throughout the study process, including data handling and reporting. All procedures were carried out in accordance with the ethical principles of the Declaration of Helsinki. 

Sampling and site selection

Purposive sampling was employed to select survey sites, taking into account geographic distribution, functional importance, and population density. The locations covered a range of categories, including educational institutions (primary schools, secondary schools, and colleges), religious places (temples and pilgrimage sites), public transport hubs (bus stands and railway stations), and hospitals and healthcare centers. In total, 50 locations were assessed during the survey period, each chosen for its high public footfall and potential for tobacco law violations.

Data collection tool

A structured observational checklist and questionnaire (see Appendix A), adapted from tools developed by the Johns Hopkins Bloomberg School of Public Health, the International Union Against Tuberculosis and Lung Disease, and the Ministry of Health and Family Welfare, was used to assess compliance with key COTPA provisions [11]. Specifically, we evaluated Section 4 (prohibition of smoking in public places), Section 5 (ban on advertisement, promotion, and sponsorship of tobacco products), Section 6a (prohibition of sale to or by minors), Section 6b (prohibition of sale within 100 yards of educational institutions), and Sections 7-9 (requirements for packaging, pictorial warnings, and product labeling). Additional observations captured the presence of prohibited designated smoking areas, 'No-Smoking/No-Tobacco' signage, ashtrays or smoking aids, tobacco sale points, packaging details, indirect advertising (e.g., branded paraphernalia), and related site characteristics.

Data collection procedure

The data were collected by trained enumerators under the supervision of a public health professional. Enumerators received training on COTPA provisions, observational protocols, and ethical considerations. Each site was systematically observed using the checklist and questionnaire, covering both indoor and outdoor areas where relevant. Observations recorded included site type, address, date and time of visit, evidence of smoking or sales, signage display, age-verification practices, packaging warnings, and photographic evidence where possible. Visits were scheduled during peak operational hours (e.g., office timings, prayer hours, or busy market periods) to capture realistic compliance levels.

Data entry and analysis

Data was captured using the printed questionnaire (see Appendix A) and was entered into Microsoft Excel (Microsoft Corp., Redmond, WA, USA). Compliance indicators were coded as 1 = yes (compliant), 0 = no (non-compliant), and 8 = not applicable (e.g., Section 6b is not applicable for sites other than educational facilities). Information was analyzed using descriptive statistics, with compliance expressed as frequencies and percentages for each section of the COTPA. Additionally, 95% CI was estimated and reported for overall non-compliance across the various sections assessed, ensuring greater precision in the findings.

## Results

Of the total sites, 10 (20.0%) were health facilities, 23 (46.0%) were educational facilities, which included 19 (38.0%) primary and secondary schools and four (8.0%) colleges and universities, seven (14.0%) sites were places of worship, one (2.0%) was a fitness center or sports facility, and one (2.0%) was a government office building. Recreation parks and bus terminals, train stations, or bus shelters each accounted for three (6.0%) sites, while two (4.0%) sites were libraries (Table 1).

**Table 1:** The type of public sites assessed for compliance with the COTPA (total n=50) COTPA: Cigarettes and Other Tobacco Products Act

Type of site assessed	Values, n (%)
Health facility	10 (20.0)
Education facility	23 (46.0)
Primary & Secondary	19 (38.0)
College & University	4 (8.0)
Place of worship	7 (14.0)
Fitness center/Sports facility	1 (2.0)
Government office building	1 (2.0)
Recreation park	3 (6.0)
Bus terminal, train station, bus shelter	3 (6.0)
Library	2 (4.0)

Among the sites assessed for compliance with the COTPA, a total of 26/50 (52.0%) were non-compliant with Section 4, 3/50 (6%) with Section 5, and 8/50 (16.0%) with Section 6a. Non-compliance with Section 4 was observed in 6/10 (60.0%) health facilities, 9/23 (39.1%) educational facilities, including 7/19 (36.8%) schools and 2/4 (50.0%) colleges or universities, 5/7 (71.4%) places of worship, 1/1 (100.0%) government office building, 3/3 (100.0%) bus terminals or transport stations, and 2/2 (100.0%) libraries. In all these sites where smoking was seen in public places, it was observed outside the premises, except in bus terminals or transport stations, where it was present both outside and inside the sites assessed. For Section 5, non-compliance was recorded in 3/50 (6%) sites, namely 1/23 (4.3%) educational facilities (a college or university), 1/7 (14.3%) places of worship, and 1/3 (33.3%) transport hubs. Of the total three instances of advertisement of tobacco products, only the place of worship featured direct advertisement, while the rest had both direct and indirect advertisements. With respect to Section 6a, non-compliance was noted in 3/23 (13.0%) educational facilities, including 1/19 (5.2%) schools and 2/4 (50.0%) colleges or universities, 2/7 (28.5%) places of worship, and 3/3 (100.0%) bus terminals or transport stations (Table 2).

**Table 2:** The type of public sites assessed for compliance with Sections 4, 5, and 6a of the COTPA (total n=50) COTPA: Cigarettes and Other Tobacco Products Act

Type of site assessed	Non-compliance with the sections of the COTPA Act assessed; n (%)		
	Section 4	Section 5	Section 6a
Health facility (n=10)	6 (60.0)	0 (0.0)	0 (0.0)
Education facility (n=23)	9 (39.1)	1 (4.3)	3 (13.0)
Primary & Secondary (n=19)	7 (36.8)	0 (0.0)	1 (5.2)
College & University (n=4)	2 (50.0)	1 (25.0)	2 (50.0)
Place of worship (n=7)	5 (71.4)	1 (14.3)	2 (28.5)
Government office building (n=1)	1 (100.0)	0 (0.0)	0 (0.0)
Bus terminal, train station, bus shelter (n=3)	3 (100.0)	1 (33.3)	3 (100.0)
Library (n=2)	2 (100.0)	0 (0.0)	0 (0.0)
Total (n=50)	26 (52.0)	3 (6.0)	8 (16.0)
95% CI	38.2-65.5	1.5-15.5	7.7-28.1

The type of public sites assessed for the presence of 'No Tobacco' signboards, both inside and outside the sites, and their compliance with Section 7.1 of the COTPA, is summarized in Table 3. A total of 28/50 (56.0%) sites were selling tobacco products, with the highest numbers in health facilities (7/10 (70.0%)), followed by educational facilities (9/23 (39.1%)), places of worship (5/7 (71.4%)), bus terminals or transport stations (3/3 (100.0%)), and libraries (2/2 (100.0%)). Of the products sold at these 28 sites, those not bearing the specified warnings under Section 7.1 (non-compliance) were observed at 26/28 (92.8%) of the sites, and all those products were smokeless forms of tobacco products. Non-compliance with Section 7.1 was found in all educational facilities, places of worship, government offices, transport hubs, and libraries checked. Outside the premises, signboards were present in 8/50 (16.0%) sites, most frequently in educational facilities 5/23 (21.7%) and health facilities 2/10 (20.0%). Section 7.5 non-compliance was recorded in only 1/28 sites (3.6%; 95% CI: 0.2-16.4), which was a library.

**Table 3:** The type of public sites assessed for the presence of no tobacco signboards, both inside and outside the sites, in compliance with Section 7.1 of the COTPA (total n=50) COTPA: Cigarettes and Other Tobacco Products Act

Type of site assessed	Selling of tobacco products; n(%) (total n=50)	Non-complaince with Section 7.1; n (%) (total n=28)	No tobacco signboards; n(%) (total n=50)	
			Outside	Inside
Health facility (n=10)	7 (70.0)	6 (85.7)	2 (20.0)	1 (10.0)
Education facility (n=23)	9 (39.1)	9 (100.0)	5 (21.7)	5 (21.7)
Primary & Secondary (n=19)	7 (36.8)	7 (100.0)	5 (26.3)	5 (26.3)
College & University (n=4)	2 (50.0)	2 (100.0)	0 (0.0)	0 (0.0)
Place of worship (n=7)	5 (71.4)	5 (100.0)	1 (14.3)	1 (14.3)
Government office building (n=1)	0 (0.0)	0 (0.0)	0 (0.0)	0 (0.0)
Health facility (n=10)	1 (100.0)	1 (100.0)	0 (0.0)	0 (0.0)
Recreation park (n=3)	1 (33.3)	0 (0.0)	0 (0.0)	0 (0.0)
Bus terminal, train station, bus shelter (n=3)	3 (100.0)	3 (100.0)	0 (0.0)	0 (0.0)
Library (n=2)	2 (100.0)	2 (100.0)	0 (0.0)	0 (0.0)
Total (n=50)	28 (56.0)	26 (92.8)	8 (16.0)	7 (14.0)
95% CI	42 – 69.1	78.3 – 98.8	7.7 – 28.1	6.3 – 25.7

The non-compliance with different sections of the COTPA among educational institutions (n=24) was assessed according to their eligibility and is presented in Table 4. For Section 4, relating to the prohibition of smoking in public places, 10/24 (41.7%) institutions were non-compliant. Under Section 5, which restricts direct or indirect advertisement of tobacco products, only one (4.2%) of the 24 institutions was non-compliant. With respect to Section 6a, prohibiting the sale of tobacco products to or by minors, 3/24 (12.5%) institutions were non-compliant. For Section 6b, prohibiting the sale of tobacco products within 100 yards of an educational institution, 10/24 (41.7%) institutions were non-compliant. Among the nine institutions eligible for Section 7.1, concerning the sale or distribution of tobacco products without specific warnings, 9/9 (100.0%) were non-compliant. Among the nine eligible sites for Sections 7.5, 8.1, and 9.2, 0/9 (0.0%) were non-compliant.

**Table 4:** Non-compliance among educational institutions assessed as per the various sections of the COTPA (total n=24) COTPA: Cigarettes and Other Tobacco Products Act

Sections of the COTPA assessed	Eligible total sites	Non-compliant n (%)
4	24	10 (41.7)
5	24	1 (4.2)
6a	24	3 (12.5)
6b	24	10 (41.7)
7.1	9	9 (100.0)

Overall, non-compliance was highest for Section 4 (26/50, 52.0%), followed by Section 6b (10/24, 41.7%). Sections 6a and 5 had lower non-compliance at 8/50 (16.0%) and 3/50 (6.0%), respectively, while Section 7.5 showed 1/28 (3.6%) non-compliance. No violations were observed for Sections 8.1 and 9.2 (0/9, 0.0%, respectively).

## Discussion

The present study highlights significant gaps in the implementation of the COTPA across multiple public sites. Overall, non-compliance was highest for Section 4 (26/50, 52%), followed by Section 6b (10/24, 41.7%), while Sections 6a (8/50, 16.0%), 5 (3/50, 6.0%), and 7.5 (1/28, 3.6%) showed comparatively lower violation rates. These findings, while broadly consistent with previous research, reveal important contextual differences when compared with studies conducted across diverse Indian settings and globally.

Compliance with Section 4 (prohibition of smoking in public places)

In our study, more than half the sites were non-compliant with Section 4, with particularly high violations in transport hubs, libraries, and places of worship. This aligns with the findings of Tripathi et al. in a South Indian city [12], where active smoking was observed in 37.5% of public places, and Chaudhary et al. in Shimla [9], where complete smoke-free compliance had not been achieved. Similarly, Ali et al.'s study in Delhi [13], Moturu et al.'s in a southeastern city [14], and Choudhary et al.'s in Rajasthan also reported partial adherence, highlighting persistent enforcement gaps [15]. Conversely, better compliance was noted in controlled settings such as tertiary health institutions in Shimla (Thakur et al.) and Srinagar (Raina et al.), suggesting that institutionalized settings may be easier to regulate [16,17]. Internationally, Anderson et al. emphasized that smoke-free policies suffer from variable compliance globally, consistent with our findings [18].

Compliance with Section 5 (prohibition of advertisement of tobacco products)

Our study found that only three (6.0%) sites were non-compliant with Section 5, lower than reported in several earlier works. Panigrahi and Sharma found widespread violations of packaging and labeling rules in Bhubaneswar slums [19], while Dinakar et al. reported poor adherence in smokeless tobacco packaging in Mangaluru [20]. Similarly, Gadiyar et al. documented weak compliance with pictorial health warnings in Goa [21]. On the other hand, Goel et al. found significant improvement post-intervention across three states [22], and Borle et al. demonstrated substantial compliance after the implementation of the Third Amendment Rules [23]. The relatively low non-compliance in our study suggests that overt advertising in formal public sites is decreasing, though covert and surrogate advertisements at points of sale may persist, as highlighted by Mallya et al. [24].

Compliance with Section 6a (sale to/by minors)

Non-compliance with Section 6a was observed in eight (16.0%) sites in our study, lower than in some previous assessments. Pradhan et al. reported substantial violations in educational institutions across India [7], while Mathias et al. documented frequent sales near Mangalore schools [25]. Nazar et al. also noted gaps in compliance with tobacco-free school guidelines in Karnataka [26]. In contrast, Mallya et al. highlighted that most vendors were aware of restrictions, though enforcement remained weak [24]. Collectively, these findings suggest that although awareness exists, practical enforcement of restrictions on sales to minors remains inadequate.

Compliance with Section 6b (prohibition of sales within 100 yards of educational institutions)

Our study recorded nearly 10 (41.7%) non-compliances under Section 6b, comparable to findings by Mathias et al. in Dakshina Kannada [25], Nazar et al. in Udupi [26], and Pradhan et al. nationally [7], all of whom reported high rates of tobacco sales in proximity to schools. Bagga and Kaur, in their two-decade evaluation of the COTPA, also identified Section 6b as one of the most challenging provisions to enforce [27]. Persistent violations across states underscore the difficulty of regulating informal vendors and require stronger local-level enforcement.

Compliance with Section 7 and related packaging/labeling provisions

Section 7 non-compliance in our study was alarmingly high, particularly for Section 7.1, where 100% of eligible educational institutions were found non-compliant. Dinakar et al. in Mangaluru [20], Gadiyar et al. in Goa [21], and Mullapudi et al. in Karnataka all reported poor compliance with health warnings, especially in smokeless tobacco and foreign brands [28,29]. Borle et al. noted improvements following regulatory amendments, though challenges persisted [23]. Our findings are consistent with these observations, reaffirming that packaging and labeling rules remain one of the weakest-enforced components of the COTPA.

The systematic review and meta-analysis by Deshmukh et al. synthesized national-level data and confirmed wide heterogeneity in compliance across different COTPA sections, with Section 4 and Section 6b being consistently weak [6]. Bagga and Kaur further identified enforcement, lack of monitoring, and vendor resistance as key systemic challenges [27]. Globally, Anderson et al. emphasized that policy compliance is the most critical determinant of tobacco control effectiveness, a lesson echoed in the Indian context [18].

Taken together, our findings resonate strongly with previous literature. Sections 4 and 6b remain the most poorly implemented, requiring targeted enforcement. Section 5 compliance appears to be improving, particularly in formal urban settings. Section 6a violations continue, though to a lesser extent than in earlier years. Section 7 remains a persistent weak spot, especially for smokeless tobacco and in educational contexts. The comparative evidence from multiple states, as highlighted by Goel et al. [10,22], Moturu et al. [14], and Choudhary et al. [9,15], indicates that while regional progress exists, national enforcement mechanisms remain inconsistent. Our findings underscore the urgent need for capacity building of local enforcement officers, vendor licensing as suggested by Mallya et al., and stronger community engagement to achieve sustained compliance [24].

Strengths and limitations

This study covered a wide range of public places and assessed compliance across multiple sections of the COTPA, offering a comprehensive snapshot of enforcement in real-world settings. The section-wise analysis adds clarity and allows meaningful comparison with national evidence. Inclusion of both indoor and outdoor areas strengthens the validity of observations. In addition, the focus on under-studied sites such as libraries, places of worship, and transport stations provides novel insights often overlooked in earlier compliance studies.

However, being cross-sectional, the findings represent only a single time point and may not reflect seasonal or enforcement-related variations. The limited geographic scope and sample size may affect generalizability. Observational methods may have missed covert violations and product-specific differences or detailed signage characteristics that were not systematically assessed.

## Conclusions

This study highlights suboptimal compliance with key provisions of the COTPA across diverse public settings. While some progress is evident, especially in restricted environments like health facilities and educational institutions, violations remain frequent in open and community-based sites such as transport hubs, libraries, and places of worship. These gaps suggest that enforcement has been uneven and insufficient to achieve the intended public health impact.

To strengthen compliance, routine monitoring and stricter enforcement drives are needed, particularly in high-footfall public spaces. Awareness campaigns targeting both the public and key stakeholders, such as vendors, transport authorities, and religious institutions, can further enhance adherence. Given the substantial influx of devotees during the pilgrimage season (e.g., Shrawani Mela at Baba Baidyanath Dham in Deoghar), pre-pilgrimage and on-site interventions such as vendor sensitization workshops, public announcements at railway stations and bus stands, tobacco-free signage near temple premises, and mobile awareness units can be implemented to maintain compliance. Integrating tobacco control activities with existing health and community programs, improving the visibility and quality of signage, and promoting community-based vigilance mechanisms could further sustain adherence. Future studies should assess product-specific violations and evaluate the effectiveness of these seasonal enforcement and awareness strategies to guide policy refinements.

## Figures and Tables

**Figure 1:**
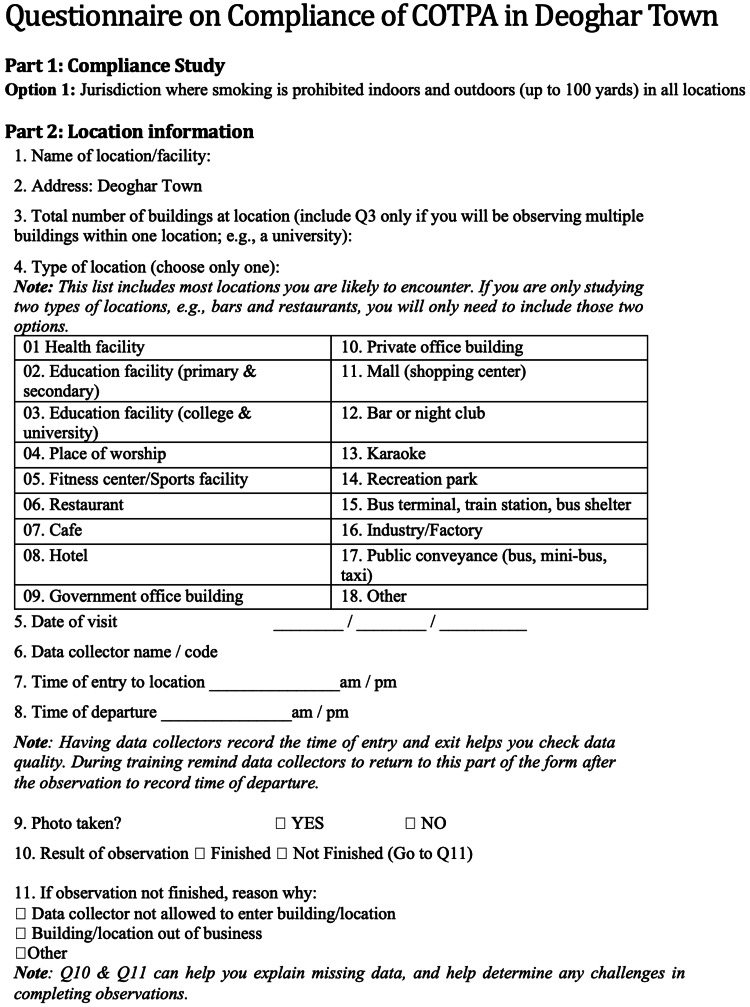
Part one and two of the questionnaire COTPA: Cigarettes and Other Tobacco Products Act

**Figure 2:**
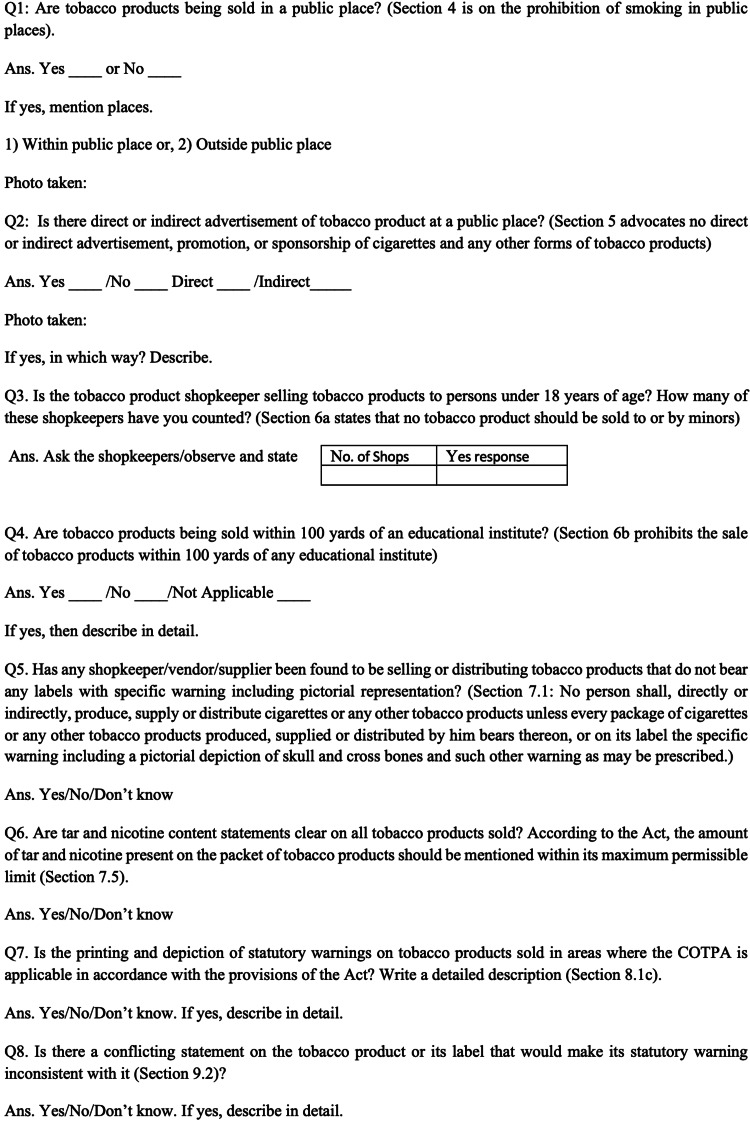
Questions from part two of the questionnaire to help gather data for the study

**Figure 3:**
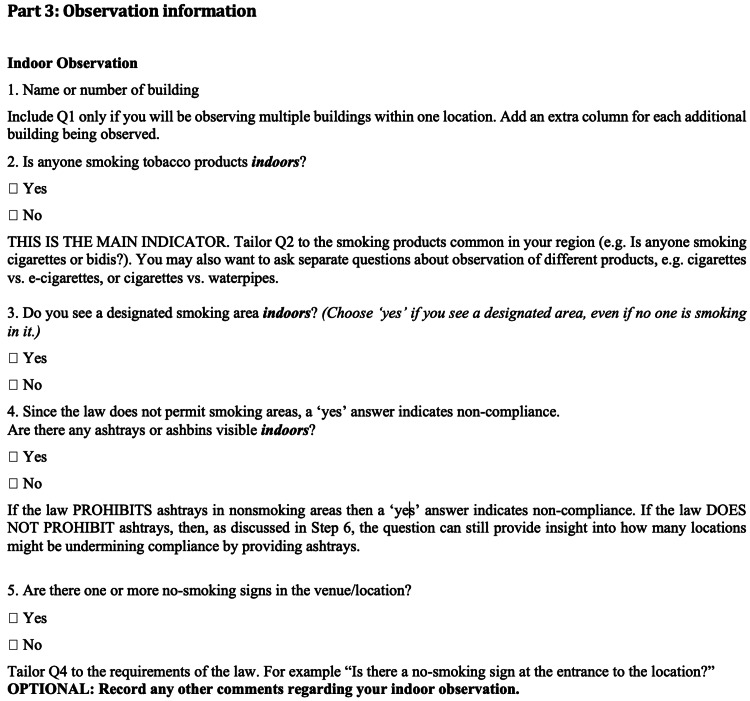
Part three of the questionnaire on indoor observations

**Figure 4:**
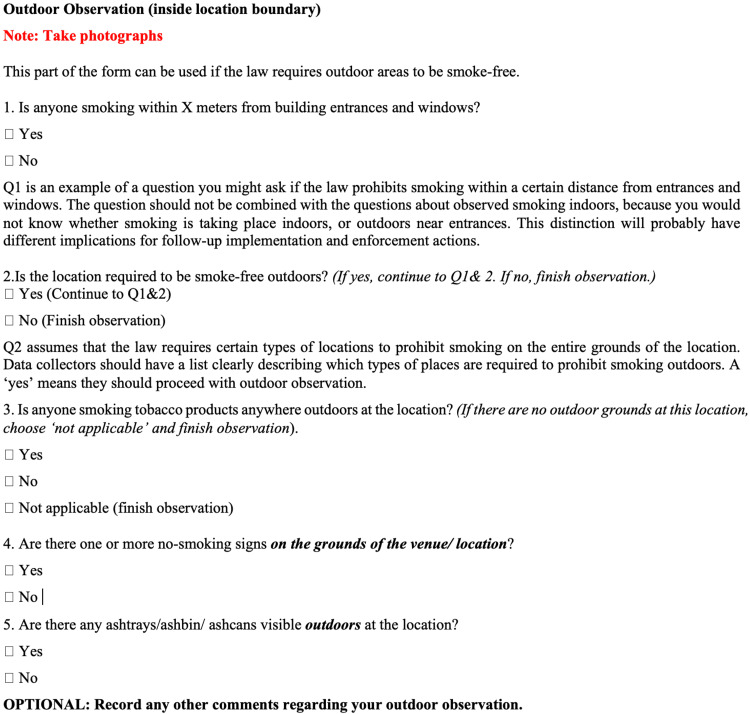
Part three of the questionnaire on outdoor observations

## Appendices

Appendix A

Data for this study were gathered via the structured, observational checklist and questionnaire featured in Figures 1-4. It was adapted from tools developed by the Johns Hopkins Bloomberg School of Public Health, the International Union Against Tuberculosis and Lung Disease, and the Ministry of Health and Family Welfare [11].
